# Beyond the Individual: The Dyadic Association Between Spousal Perceived Discrimination and Depressive Symptoms

**DOI:** 10.1093/geroni/igaf122.2930

**Published:** 2025-12-31

**Authors:** Soohyeon Ko

**Affiliations:** Harvard University, Boston, Massachusetts, United States

## Abstract

Perceived discrimination increases psychological distress across the life course. However, little is known about whether one spouse’s perceived discrimination similarly affects the depressive symptoms of the other spouse and whether this effect varies by gender or closeness to the spouse. Using data from 6,146 older couples (12,292 individuals aged 60 years or older) in the first wave of the Longitudinal Aging Study in India (2017–2018), we applied multilevel regression models to examine the dyadic associations between perceived discrimination and depressive symptoms (measured by the CES-D) in both oneself and one’s partner. We further explored whether this crossover association differs by gender and perceived closeness to one’s spouse. Results indicate that after accounting for covariates, perceived discrimination is associated with increased depressive symptoms not only in the individual (Men: β = 0.14, p < 0.001; Women: β = 0.16, p < 0.001) but also in their partner (Men: β = 0.08, p < 0.001; Women: β = 0.09, p < 0.001). Interaction analyses reveal that closeness to one’s spouse intensifies the partner effect of perceived discrimination for women (β = 0.07, p = 0.03), whereas no such effect is observed for men. These findings highlight the dyadic implications of perceived discrimination for both partners. Specifically, women who perceive greater closeness to their spouse experience a stronger crossover effect of their partner’s perceived discrimination on their own depressive symptoms.

